# Woorara as a Remedy for Traumatic Tetanus

**Published:** 1859-12

**Authors:** 


### EDITORIAL.

### WOORARA AS A REMEDY FOR TRAUMATIC TETANUS.

We notice that M. Vella, through M. Bernard, has presented to the Academy of Sciences, Paris, a paper on this subject. M. Vella had concluded that the action of the woorara is antagonistic to that of the cause of tetanus. He accordingly applied it to some of the wounds of patients affected with tetanus, and was of the opinion that the spasms were in a measure controlled by it.

While we approve the effort of M. Vella to search for new remedies against this intractable disease, we cannot adopt either his theory or his practice in this instance. Woorara was, during the latter part of the last century, tried for tetanus in London, without success. Having noticed this fact in an old journal, which is not at hand, we tried it as an antidote to strychnia, but found that mixing the two poisons together killed much more quickly than the employment of either singly. We have tried it also upon ulcers. It is, when mixed with simple cerate in proportion of 10 grs. to the ounce, quite harmless, produces no perceptible constitutional effects, and acts as a stimulant to the sore, suppressing the discharge of pus, and causing the surface to be covered with a thin layer of fibrine.

